# Photonic Crystal Sensors Based on Porous Silicon

**DOI:** 10.3390/s130404694

**Published:** 2013-04-09

**Authors:** Claudia Pacholski

**Affiliations:** Department of New Materials and Biosystems, Max Planck Institute for Intelligent Systems, Heisenbergstr. 3, Stuttgart 70569, Germany; E-Mail: Pacholski@is.mpg.de; Tel.: +49-711-689-3620; Fax: +491-711-689-3612

**Keywords:** photonic crystal, porous silicon, sensor

## Abstract

Porous silicon has been established as an excellent sensing platform for the optical detection of hazardous chemicals and biomolecular interactions such as DNA hybridization, antigen/antibody binding, and enzymatic reactions. Its porous nature provides a high surface area within a small volume, which can be easily controlled by changing the pore sizes. As the porosity and consequently the refractive index of an etched porous silicon layer depends on the electrochemial etching conditions photonic crystals composed of multilayered porous silicon films with well-resolved and narrow optical reflectivity features can easily be obtained. The prominent optical response of the photonic crystal decreases the detection limit and therefore increases the sensitivity of porous silicon sensors in comparison to sensors utilizing Fabry-Pérot based optical transduction. Development of porous silicon photonic crystal sensors which allow for the detection of analytes by the naked eye using a simple color change or the fabrication of stacked porous silicon photonic crystals showing two distinct optical features which can be utilized for the discrimination of analytes emphasize its high application potential.

## 1. Introduction

Optical sensors for the detection and quantification of hazardous chemicals, investigation of biomolecular interactions or studies on cellular systems have been developed for decades and are still a field of extensive research (reviewed for example in Reference [1]). Label-free optical sensors use mainly surface plasmon resonance and interferometry as transduction methods whose performance complements each other. A recent study on the sensitivity of localized surface plasmon resonance (LSPR) transducers in comparison to interferometric sensors identified the superiority of LSPR based devices for the analysis of thin (several nm) analyte and recognition interfaces and emphasized the advantage of interferometric sensors for the investigation of thicker layers [2]. The high potential of porous silicon for fabrication of interferometric sensors originates from its easily controllable fabrication process resulting in layers with defined porosity (refractive index), its high surface area, simple surface chemistry, and full compatibility with microprocessing techniques. In the early stages porous silicon based sensors were composed of a single porous layer on the silicon substrate leading to Fabry-Pérot interference. Here, the reflectivity spectrum shows interference fringes which correspond to constructive and destructive interference from light reflected at the air/porous silicon and porous silicon/crystalline silicon interfaces. Changes in the average refractive index of the porous silicon layer caused by infiltration or adsorption of analytes are detected by spectral shifts in the reflectivity spectrum [3,4].

**Figure 1.**
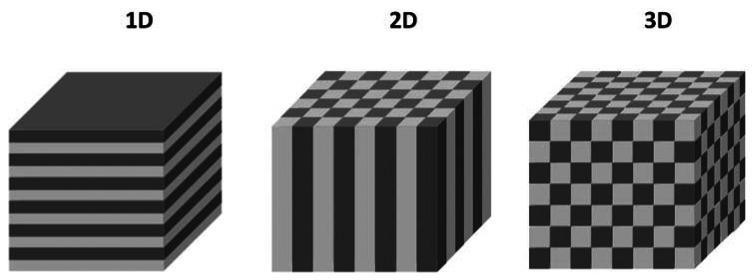
Photonic crystals (adapted from Reference [7]).

Photonic crystals are composed of alternating regions of high and low dielectric constants and can be obtained in 1, 2, or 3 dimensional periodic array arrangements (Figure 1). By choosing appropriate dielectric constants and geometry these materials can exhibit a photonic band gap (stop band) which is characterized by the prevention of light propagation at a range of frequencies defined by the internal structure of the photonic crystal. Hence, photonic crystals can be employed as optical filters and allow for the isolation of narrow reflection bands. Since the conceptual introduction of photonic crystals by Yablonovitch and John in 1987 [5,6] photonic crystals have been fabricated from diverse materials including semiconductors, polymers, oxides, and porous silicon.

The implementation of more complicated optical structures, e.g., photonic crystals, into porous silicon based sensors improved their sensing capabilities in two ways. On the one hand the sensitivity and specificity provided by the porous silicon sensor was considerable enhanced. The sharp resonant optical response of the photonic crystal makes it much easier to detect small shifts in the reflectivity spectrum leading to detection limits on the femtomolar level. The incorporation of a lateral porosity gradient provides a size exclusion filter resulting in improved specificity of a porous sensor [8]. On the other hand photonic crystal sensors allow for the detection of analytes by the naked eye. Based on their internal structure photonic crystal solely reflect light at distinct frequencies and therefore appear as a pure color to the eye. Penetration of analytes into the pores consequently cause easily noticeable color changes in the photonic crystal sensors.

## 2. Fabrication of Porous Silicon Photonic Crystals

**Figure 2.**
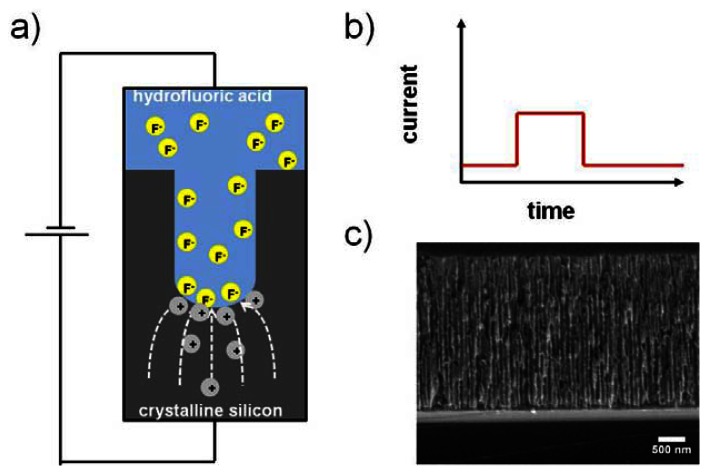
Fabrication of porous silicon. (**a**) Schematic of porous silicon formation by electrochemical etching. Adapted from Reference [11]. (**b**) Applied current density versus time waveform and corresponding SEM image of etched a porous silicon layer (**c**).

Porous silicon was accidently discovered in the mid-1950s by Uhlir and Uhlir, who tried to find a convenient method for electropolishing silicon wafers [9]. They found that upon electrochemical etching of silicon wafers in fluoride containing solutions small holes can propagate in the <100> direction in the Si wafer. The overall electrochemical reaction for Si etching is given by Equation (1):

(1) $$Si+6\;F^{–}+2\;H^{+}+2\;h^{+}\to {{\text{SiF}}_{6}}^{2-}+H_{2}$$

in which h^+^ is a hole injected into the valence band of the semiconductor. The simplicity of this reaction equation belies the complexity of porous silicon formation which involves electronic as well as chemical factors. Numerous parameters such as the applied voltage, the chosen silicon substrate (dopant type and concentration), the electrolyte composition, temperature and light intensity have a considerable influence on the resulting silicon nanostructure. A detailed discussion of porous silicon formation is beyond the scope of this review and can be found in reference [10]. However, in general pores nucleate randomly but homogenously on the silicon surface upon electrochemical etching leading to pores with a narrow pore diameter distribution. The pore diameters can be easily controlled and varied between a few and several thousands of nanometers. Figure 2(a) shows a schematic of the porous silicon formation process. Etching occurs mainly at the pore tips as holes are directed to the tips by the electric field and etching of the pore walls is prevented by passivation upon etching. Hence, dissolution of silicon is primarily obtained at the porous silicon/crystalline silicon interface. An example for an applied current density versus time waveform for electrochemical etching and a corresponding SEM image of an etched porous silicon layer are displayed in Figure 2(b,c), respectively.

### 2.1. 1D Photonic Crystals

**Figure 3.**
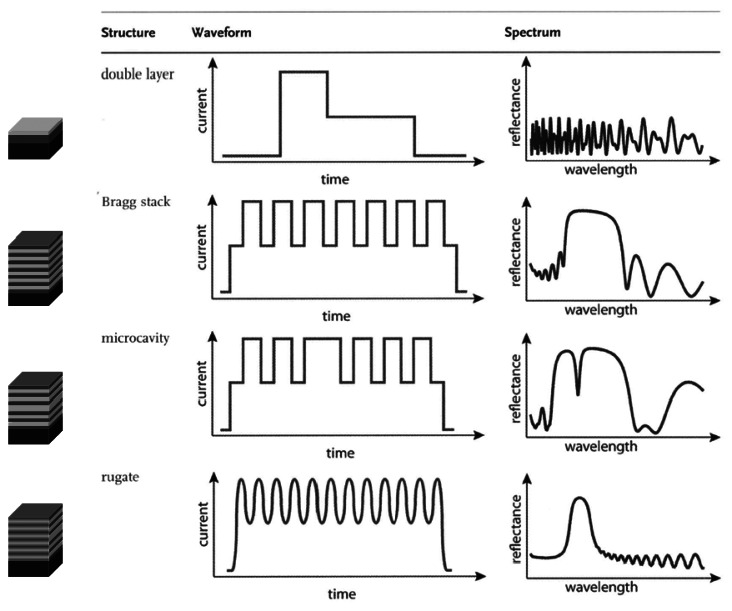
Porous silicon based 1D photonic crystals. Reprinted from Reference [13] with permission. Copyright Wiley-VCH 2012.

The first porous silicon based photonic crystal was electrochemically etched into a silicon wafer by Vincent in 1994 [12]. The key to its successful fabrication was the observation that the amount of silicon dissolved at the interface of the pores and the crystalline silicon is directly related to the current passed at that moment. Thus, changes in the applied current result in corresponding changes in the porosity of the formed porous silicon leading to porous silicon layers possessing different refractive indices. Vincent varied the applied current between two values leading to alternating discrete porous silicon layers of two different refractive indices. By carefully choosing appropriate porosities and thicknesses of the porous silicon layers he was able to create a Bragg reflector which provides a photonic bandgap rejection of a wide range of wavelengths of light. This band is called the stop band of the structure and its position is defined by Equation (2):

(2) $$\lambda_{SB}=4n_{i}L_{i}$$

in which the product nL represents the optical thickness which is composed of the refractive index (n) and the thickness (L) of the porous silicon layer. A microcavity is composed of a Fabry Pérot cavity sandwiched between two Bragg reflectors. Thereby the periodicity of the refractive index profile is disturbed and a sharp resonant dip in the stop band is obtained. In contrast to the abrupt changes between two refractive indices in Bragg stacks the refractive index profil in rugate filters is characterized by a smooth, sinusoidal variation in the refractive index. Figure 3 summarizes the presented 1D photonic crystal structures prepared with porous silicon including the approximate current density waveforms and representative spectra.

### 2.2. 2D and 3D Photonic Crystals

**Figure 4.**
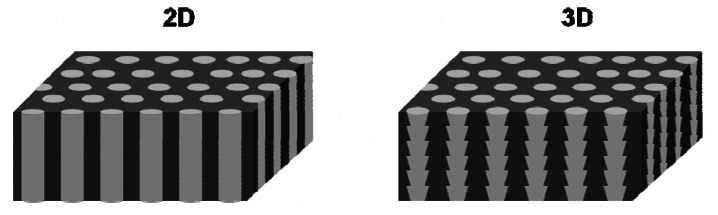
Exemplary schematics of 2D and 3D porous silicon photonic crystals.

2D photonic crystals are often realized either by a square lattice of rods with high dielectric constant which are embedded in a medium with a low dielectric constant or by a hexagonal lattice of holes with a low dielectric constant surrounded by a material with a high dielectric constant [14]. Lehmann *et al.* electrochemically etched ordered macropores into silicon and thereby fabricated structures which showed a complete 2D bandgap in the near-infrared for the first time [15]. Macroporous silicon is in general obtained by pre-patterning an n-type silicon wafer using standard lithographic methods followed by electrochemical etching in hydrofluoric acid containing solutions under backside illumination. The illumination generates electronic holes which promote the dissolution of silicon at the pore tips leading to the formation of straight pores with very high aspect ratio [16,17]. The pore arrangement in x, y direction is defined by the lithographic mask. The diameter of the pores in depth (z direction) can be varied by changing the illumination intensity, the applied current density and by post-treatment of 2D structures with basic solutions. Thereby 3D photonic crystals based on macroporous silicon were fabricated [18–21]. In Figure 4 schematics of 2D and 3D porous silicon photonic crystals are displayed. Other approaches to 2D and 3D photonic crystals include the utilization of porous silicon as low dielectric constant material in hexagonal arrays or as sacrificial layer for the generation of more complicated structures in silicon [22,23].

## 3. Optical Sensors

Porous silicon films on crystalline silicon show a so-called Fabry-Pérot fringe pattern in their reflectivity spectrum which is caused by constructive and destructive interference of reflected light rays at the interfaces bordering the porous layer. The wavelengths of the fringe maxima λ_max_ in the reflectivity spectra can be calculated by using Equation (3):

(3) $$m\lambda_{\max}=2nL$$

where m is an integer corresponding to the spectral order of the fringe, n is the average refractive index of the porous layer and L is its thickness [24]. Optical sensors based on porous silicon mainly detect analytes by shifts of the fringe pattern on the wavelength scale resulting from changes in the average refractive index of the porous layer. For this purpose the effective optical thickness (EOT, 2 nL) is often estimated by applying a Fourier transform to a plot of reflected light intensity versus frequency. The position of the resulting single peak in the Fourier transform corresponds to the effective optical thickness of the porous layer. Here, the refractive index n is a composite index which is composed of the refractive index of the medium filling the pores and the refractive index of the material from which the porous structure has been created (Si).

**Figure 5.**
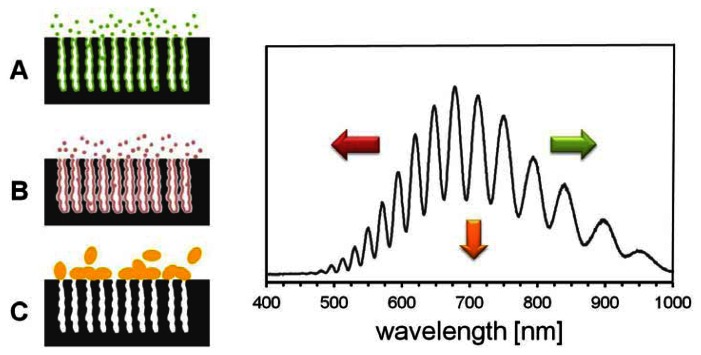
Optical sensors based on porous silicon. (**A**) Increase of the average refractive index of the porous layer leads to a shift of the interference pattern to longer wavelengths. (**B**) If the average refractive index of the porous layer decreases the fringe pattern in the reflectivity spectrum shifts to shorter wavelengths. (**C**) Adsorption or binding of the analyte to the medium/porous silicon interphase changes the refractive index contrast and decreases the amount of reflected light.

Reflectometric sensing modes which have been reported for porous silicon based sensors are summarized in Figure 5. If the refractive index of the medium filling the pores increases, the fringe pattern in the reflectivity spectrum shifts to the red end of the spectrum (longer wavelengths) and consequently to higher EOT values (Figure 5(A)). This sensing mode has extensively been employed for the detection of hazardous chemicals and biomolecular recognition events. A blue shift (shorter wavelengths) of the interference pattern is obtained if the average refractive index of the porous layer decreases (Figure 5(B)). The decrease in refractive index was correlated to the corrosion of the porous silicon caused by analytes such as negatively charged DNA and transition metal complexes. Finally, changes in the amount of reflected light resulting from changes in the refractive index contrast at the porous silicon layer interfaces can be used for the detection of analytes which do not penetrate the porous layer (Figure 5(C)). All of the described reflectometric sensing modes for single-layered porous silicon can be transferred to multi-layer porous structures whose more elaborate optical properties increase the sensitivity of the porous silicon sensor and allow for background correction [13].

### 3.1. Stabilization and Functionalization of Porous Silicon

The surface chemistry of porous silicon is governed by the reactivity of silicon-silicon and silicon-hydrogen bonds which are formed upon electrochemical etching. Both species can react with atmospheric oxygen and water at ambient conditions leading to the oxidation of the porous silicon structure. The resulting SiO_2_ nanostructure is unstable in water-based media and will especially be dissolved in aqueous solutions containing nucleophilic molecules and tensides. Hence, the porous silicon surface has to be stabilized for (bio)sensing applications. For this purpose two strategies have been developed resulting in the formation of either silicon-oxygen of silicon-carbon bonds (reviewed in [25,26]). Silicon-oxygen bonds are often generated by thermal oxidation in air, ozone oxidation, and oxidation by organic molecules. The oxidized silicon surface presents silanol groups which can be utilized for further functionalization reactions including the introduction of amines and subsequent coupling of biomolecules to the porous silicon surface via the formation of amide bonds. However, the porous SiO_2_ structure remains prone to degradation in aqueous media despite its functionalization with silanes as all layers containing silicon-oxygen bonds are susceptible to hydrolysis at high pH (higher than pH 7) in general. A more stable surface functionalization was achieved by the formation of silicon carbon bonds. Based on the low electronegativity of carbon the nucleophilic attack of silicon carbon bonds by water or hydroxide is hampered. Silicon-carbon bonds are formed by hydrosilylation, chemical or electrochemical grafting techniques as well as carbonization reactions. Notwithstanding the obvious advantages of porous silicon stabilized by silicon-carbon bonds its application was neglected several years due to its tedious fabrication in oxygen excluding conditions (inert atmosphere).

### 3.2. 1D Photonic Crystal Sensors

#### 3.2.1. Gas Sensing

Nanoporous material is able to concentrate vapor by microcapillary condensation. This phenomenon can be described using the Kelvin Equation (4) which relates the pore radius r to the partial pressure at which condensation of vapor will be observed at a defined temperature [27]:

(4) $$r=\frac{\gamma^{V}}{RT\ln(P/P^{0})}$$

Here, γ represents the surface tension at the gas/liquid interface, V represents the molar volume of the liquid, R represents the gas constant, P is the observed vapor pressure and P^0^ the pressure of the vapor at the given temperature T. This relation emphasizes the suitability of porous silicon sensors for the detection of vapors such as volatile organic compounds (VOCs), organophosphate nerve agents, ammonia, and hydrofluoric acid [28]. Zangooie *et al.*, stimulated the following intensive research on optical porous silicon gas sensors in 1997 by studying the optical changes in thin porous layers caused by vapor exposure using spectroscopic ellipsometry [29]. Two years later Snow *et al.* [30] reported on drastic changes in the reflectivity spectra of porous silicon 1D photonic crystals resulting from exposure to volatile VOCs. A Bragg mirror was fabricated by electrochemical etching of silicon and the shift of the reflectivity peak on the wavelength scale over time was monitored. The experiments showed that different organic vapors, e.g., chlorobenzene and acetone, lead to distinct shifts of the reflectivity peak which can be correlated to their refractive index. The results confirmed the condensation of vapor in the pores of the employed porous silicon sensor. A challenging task of until then developed porous silicon gas sensors was the discrimination between vapors of substances with similar refractive indices. Mulloni and Pavesi addressed this issue by the introduction of an optical sensor which detects two different optical properties at the same time [31]. For this purpose a microcavity structure was etched into lightly doped silicon which additionally shows high luminescence efficiency. Exposure of this sensor to organic solvents led to large redshifts of the resonance peak of the microcavity which were attributed to changes in the composite refractive index of the porous layers. At the same time luminescence intensities were measured and correlated with differences in the dielectric functions of the analytes. Thereby an increased specificity and sensitivity of porous silicon based gas sensors for VOCs was achieved. This concept was extended to the fabrication of a porous silicon microcavity sensor which monitored three different parameters, *i.e.*, the resonance wavelength of the microcavity, the luminescence, and the electrical conductance [32]. However, the more complicated sensor lay-out of the multi-parametric sensors and the encouraging performance of porous silicon microcavity sensors detecting only changes in the resonance wavelength pushed the research on the latter in the following years. De Stefano *et al.*, investigated systematically the response of porous silicon microcavity structures on exposure to flammable substances [33]. A deeper insight into capillary condensation in porous silicon optical microcavities was gained by time-resolved sensing of iso-propanol [34]. The experimental results as well as the corresponding numerical simulations of changes in the position of the cavity peak on the wavelength scale demonstrated an immediate and homogenous filling of the pores. Similar behavior was reported for porous silicon rugate structures [35,36]. By removing the porous silicon film from the silicon substrate using ultrasonication microparticles referred to as “smart dust particles” were obtained which were successfully employed for remote sensing of chemicals [37]. An even more practical application was suggested by King *et al.*, who glued a porous silion rugate film on top of an optical fiber and used this sensor for monitoring VOCs breakthrough in an activated carbon filtration bed [38].

Pretty soon the importance of the surface chemistry of the porous silicon based sensors for its gas sensing performance was noticed and investigated. Chapron *et al.*, fabricated a porous silicon rugate filter whose reflectivity peak was located in the infra-red spectral range and studied the influence of surface modifications such as chemical oxidation and hydrosilylation with octadecene on the optical response of the porous silicon sensor [39]. Only small differences in the sensitivity of the two rugate sensors were observed. However, a thorough analysis of the reflectivity spectra allowed for a quantitative determination of the porosity as well as the thickness of the SiO_2_ and alkyl layer formed on the porous silicon surface. A systematic study on this topic was later published by Ruminski *et al.*, who demonstrated a strong impact of the surface chemistry on the sensitivity and stability of the porous silicon gas sensors [40]. Exposure of the sensors to either more hydrophilic (isopropanol) or hydrophobic VOCs (heptane) led to pronounced redshifts of the rugate peaks in the reflectivity spectrum which were in all cases stronger for hydrophilic chemicals expect for carbonized samples resulting from decomposition of acetylene on freshly etched porous silicon. The carbonized porous silicon showed higher sensitivity for heptane vapors. At the same time the stability of the porous silicon sensors was investigated and it turned out that especially carbonized, thermally oxidized, and dimethylsiloxyl-terminated porous silicon structures are stable for a long time qualifying them for environmental monitoring applications. Furthermore composites of porous silicon and carbon have a one order of magnitude lower detection limit for toluene in comparison to freshly etched porous silicon sensors [41]. Another strategy for increasing the sensitivity of porous silicon 1D photonic crystal sensors was reported by Jang *et al.* [42]. They incorporated copper(II) sulfate in a porous silicon Bragg mirror and used a LED as light source. By replacing the commonly used tungsten halogen lamp by an LED and monitoring changes in the reflectivity intensity instead of shifts in the Bragg peak position the detection limit of the sensor for triethyl phosphate was lowered from 1.4 ppm to 150 ppb.

**Figure 6.**
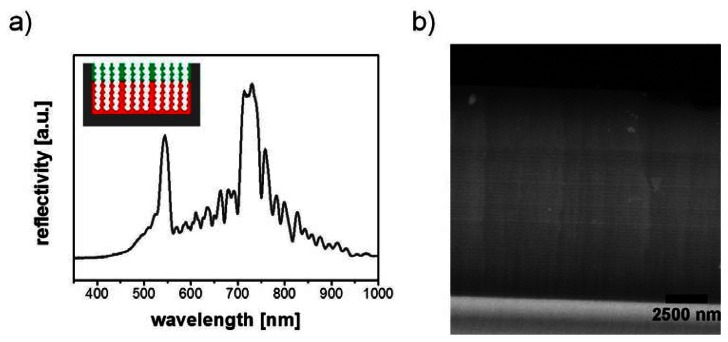
Example for a porous silicon gas sensor. (**a**) Reflectivity spectrum of a double layer rugate structure which has been utilized for the elimination of humidity based changes in the reflectivity spectrum interfering with the detection of organic vapors. (**b**) Backscatter SEM image of a double layer rugate structure.

Another challenge for porous silicon based optical sensors is zero-point drift of the reflectivity spectrum caused by fluctuations in light source intensity, temperature, and humidity. In order to correct for the inevitable background noise the introduction of a reference channel directly on the porous silicon sensor platform was suggested. King *et al.* realized this reference channel by etching a porous silicon 1D photonic crystal showing two reflectivity maxima in the spectrum and infiltration of a pH sensitive probe (bromothymol blue) into its pores [43]. The absorbance maximum of the indicator dye overlapped with one of the reflectance peaks of the photonic crystal upon exposure to NH_3_ vapor resulting in a considerable intensity decrease of this reflectance peak. At the same time the second reflectance peak located in a different spectral range was unaffected. Hence, a ratio of the intensity of the two peaks can correct for experimental fluctuations and thereby increase the sensitivity of the sensor for NH_3_ vapor. The concept of an internal reference channel was also used for the detection of HF and Cl_2_[44]. An even more intriguing exploitation of this strategy was the fabrication of double stacks of rugate filters whose surfaces were functionalized with different molecules [45]. By giving the top stack hydrophobic and the bottom stack hydrophilic properties and calculating the weighted differences between the two peak frequencies of the two rugate filters a humidity-compensating sensor for VOCs was obtained. Figure 6 shows a reflectivity spectrum and a backscatter SEM image of two separate rugate structures etched on top of each other. It has to be emphasized that the stacking order of the rugate filters is important for the appearance of both rugate peaks in the reflectivity spectrum [46].

Recently, another approach for improving the specificity of porous silicon gas sensors was introduced by King *et al.*, They mounted a 1D phtonic crystal on a thermoelectric Peltier device and monitored shifts in the spectral position of the photonic stop band upon exposure to VOCs as a function of temperature [47]. The observed hysteresis loops were explained by differences in the diffusion and adsorption properties of the vapors within the porous structure and turned out to be characteristic for each investigated analyte. Kelly *et al.*, also used the parameter diffusion for the identification and quantification of VOCs with porous silicon photonic crystal sensor [48]. Here, the porous silicon sensor consisted of three stacked mesoporous rugate filters displaying three distinct peaks in the reflectivity spectrum. The layer in the middle serves as “drift tube” and delays the diffusion of the vapor into the bottom layer. The optical response of the three layers was monitored over time and the sequential response of the three layers was successfully correlated to the rate of analyte diffusion through the porous silicon structure. This method allowed for the identification of VOCs in parts per million concentrations.

#### 3.2.2. Biosensing

The first porous silicon based biosensor was fabricated in 1997 and consisted of a single porous silicon layer whose Fabry-Pérot fringes in the reflectivity spectrum shifted on the wavelength scale upon binding of biomolecules (DNA and proteins) to the functionalized porous silicon surface [3]. The performance of these single layer porous silicon sensors was compared to the sensitivity of photonic crystal structures etched into silicon, e.g., Bragg stack and microcavity, by Anderson *et al.* [49]. Changes in the reflectivity spectrum upon infiltration of sucrose solutions were theoretically calculated and experimentally measured. As expected the 1D photonic crystal structures showed higher sensitivities for refractive index changes in the pores than the single layer sensor. However, in contradiction to the theoretical predictions the Bragg stack displayed a larger redshift of the resonance peak on the wavelength scale in comparison to the microcavity structure emphasizing the importance of a well-thought-out design for the multilayer sensors. Ouyang *et al.*, systematically optimized the etching parameter for macroporous silicon microcavities in order to allow for the detection of macromolecule infiltration into the porous structure [11,50]. The sensitivity of the prepared macroporous mirocavity structures depended on several parameters such as the pore size, the porosity contrast between the porous layers, and the thickness of the layers. Thereby it was crucial to adjust the macroporous structure to the biosensing task. The excellent optical sensing properties of the developed macroporous sensor were demonstrated by infiltration of rabbit IgG and detection of biotin/streptavidin interaction with a detection limit of 0.3 ng/mm^2^. An in-depth study on this topic was published by DeLouise *et al.*, who investigated a series of microcavity biosensor devices for immobilization of glutathione-S-transferase within the porous matrix [51]. The amount of immobilized enzyme was indirectly quantified by measuring the enzyme activity and successfully cross-correlated to the magnitude of the optical response of the microcavity. Detection limits as low as 50–250 pg/mm^2^ were achieved for the optimized macroporous silicon sensors—values which could compete with standard SPR devices. Two years later, in 2007, macroporous silicon microcavity photonic bandgap sensors were used for selective and quantitative detection of protein interactions [52].

Almost all of the previously described porous silicon biosensors focused on an increase in the composite refractive index of the porous photonic crystal structure by infiltration of substances into the pores which possess higher refractive indices and consequently monitored shifts of the resonance peaks to higher wavelengths (redshift). In contrast, Kilian *et al.*, reported on peptide-modified porous silicon rugate filters for the detection of protease activity by pronounced blue shifts of the reflectivity peak [53]. Here, the high refractive index peptides were removed from the porous matrix by enzyme cleavage leading to a decrease in the composite refractive index of the porous layer. A tailor-made surface functionalization strategy allowed them to detect protease concentrations of 37 nM.

**Figure 7.**
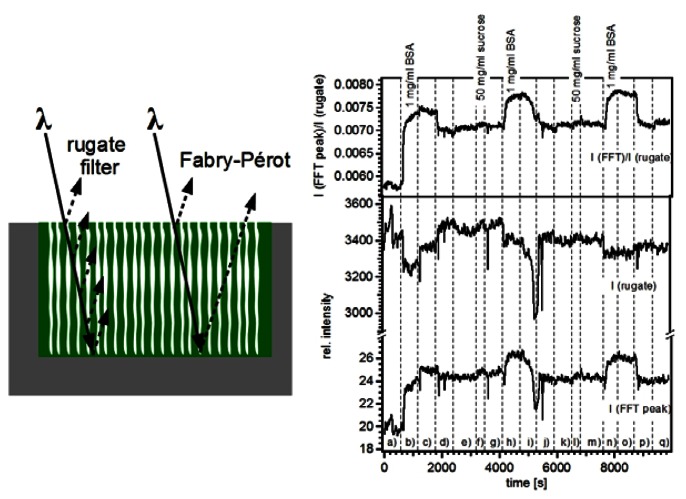
Porous silicon photonic crystal considered as optical double layers. (**a**) Schematic of a 1D porous silicon photonic crystal. Interference of light beams occurs from all layers of the multilayered structure (rugate) as well as from reflections at the interfaces bordering the porous layer (Fabry-Pérot interference). (**b**) Sensogram of the structure upon exposure to sucrose and BSA solutions. Reprinted from Reference [54] with permission. Copyright Wiley-VCH 2007.

Pacholski and Sailor monitored intensity changes in the reflectivity spectrum of a porous silicon rugate structure upon exposure to sucrose and bovine serum albumin (BSA) solutions [54]. The reflectivity spectrum of a 1D porous silicon photonic crystal consists of a strong peak corresponding to the optical response of the photonic crystal (rugate filter) and Etalon fringes (Fabry-Pérot interference) originating from reflections at the interfaces of the porous structure (Figure 7(a)). By following the intensity changes of these two optical features instead of shifts in the position of the “rugate” peak on the wavelength scale the refractive index contrast at the interface medium/porous layer was investigated rather than refractive index changes in the porous layer. In Figure 7(b) the intensity changes of the rugate peak and the intensity changes of the Fabry-Pérot (here represented by the effective optical thickness) upon analyte exposure are displayed. Both signals were not suitable for a reliable detection of BSA and sucrose. However, calculating the ratio between the intensity of the EOT and the “rugate” peak intensities led to a sensitive detection of BSA. It was concluded that the intensity of the “rugate” peak can serve as reference channel whereas the EOT intensity detects binding events to the sensor surface.

**Figure 8.**
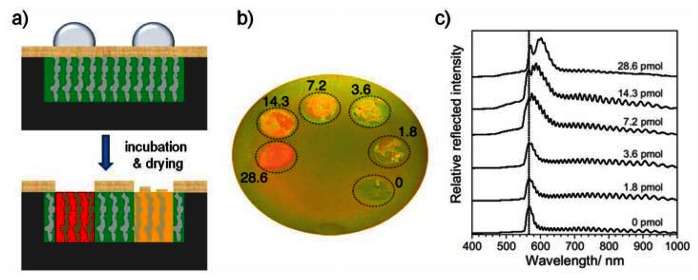
Optical detection of protease activity using a protein-coated porous silicon sensor. (**a**) Schematic of the sensor principle. A hydrophobic porous silicon rugate structure is coated with a hydrophobic protein. Addition of small amounts of proteases leads to digestion of the protein layer. The proteolytic cleavage products infiltrate into the porous structure resulting in a visible color change. (**b**) Photo of a protein-coated 1D photonic crystal which has been treated with different amounts of proteases (given in picomoles). (**c**) Reflectivity spectra taken from the spots on the protein-coated porous silicon sensor shown in (b). Parts (a) & (b) reprinted from Reference [55] with permission. Copyright Wiley-VCH 2006.

A new and exciting concept for the detection of biomolecules by porous silicon 1D photonic crystal sensors was introduced by Orosco *et al.*, in 2006 [55]. They determined protease activity by color changes that were observable by the naked eye making the use of a spectrometer for optical signal transduction dispensable. A schematic of the sensor principle is depicted in Figure 8(a). A porous silicon rugate filter was electrochemically etched. Methyl groups were grafted onto its surface in order to provide hydrophobicity and thereby prevent water penetration into the pores. After coating the sensor surface with a hydrophobic protein, small droplets of water containing different amounts of proteases were placed on the sensor surface. The proteases digested the hydrophobic protein and the proteolytic cleavage products penetrated the hydrophobic pores leading to an increase in the composite refractive index of the porous layer and consequently to a color change. Protease concentrations as low as 7.2 pmol were detectable by the naked eye (Figure 8(b)). By taking reflectivity spectra the detection limit could not be improved (Figure 8(c)). Gao *et al.*, transferred this concept to hydrophilic proteins [56]. Further development of porous silicon 1D photonic crystal sensors for visual colorimetric readout was reported by Bonanno and De Louise in 2010 [57]. Here, the integration of a stimuli-responsive hydrogel into the porous structure whose cross-linking densities is controlled by interactions with the analyte is the key to achieve direct optical detection.

A push towards commercial application of porous silicon sensors was evoked by Bonanno and DeLouise in 2010. They developed a label-free porous silicon competitive inhibition immunosensor for the rapid and reliable detection of opiates in urine [58]. Competitive binding assays enable the detection of small molecules like opiates by optical sensors which transduce refractive index changes into an optical signal. Tiny refractive index changes caused by infiltration of small molecules into the pores lead to a very small change in the composite refractive index of the porous layer. Hence, the corresponding optical response of the sensor is not sufficient for reliable sensing. By specific binding of appropriate antibodies to the analyte the change in the refractive index is considerably increased resulting in a sensitive and reproducible optical response of the sensor to the analyte. This principle has been exploited for the porous silicon competitive binding immunosensor. Here, a porous silicon 1D photonic crystal was functionalized with an opiate analogue. If this sensor was exposed to a solution of antimorphine IgG the antibody was solely bound to the immobilized opiate analogue leading to a maximum wavelength shift of the stop band peak (reference). For a mixture of a test solution containing opiates and the same amount of antibody a smaller red-shift in the biosensor signal was obtained. This observation is based on the fact that less antibody is available for binding to the sensor surface as the opiates in the test solution compete with the opiate analogue bound to the sensor surface. The performance of such a label-free porous silicon immunosensor assay was positively evaluated for the detection of opiates in a blind clinical study [59].

Not only biomolecules have been sensed by porous silicon optical sensors but also biological organisms such as bacteria, eukaryotes, and prokaryotes. First studies on this topic were performed using changes in the photoluminescence of a porous silicon microcavity as optical transduction. Tailor-made ligands for Gram-(−) bacteria were bound to the pore walls of these sensors and allowed for discrimination of Gram-(−) and Gram-(+) bacteria in lysed bacteria solutions [60,61]. In 2006 Schwartz *et al.*, investigated changes in the intensity of light scattered from a porous silicon 1D photonic crystal for monitoring morphology changes in an assembly of hepatocyte cells upon exposure to toxins [62]. The same method was employed for the detection of virus infection in bacteria [63]. To the best of my knowledge there has been no report on porous silicon photonic crystal sensors which detect whole cells by changes in their reflectivity spectrum. Indirect observation of live cells was achieved by seeding cells which secreted proteases on a biopolymer loaded porous silicon rugate filter. These enzymes digested the biopolymer incorporated in the porous layer and thereby provoked a blue-shift of the reflectivity peak [64].

### 3.3. New Concepts

**Figure 9.**
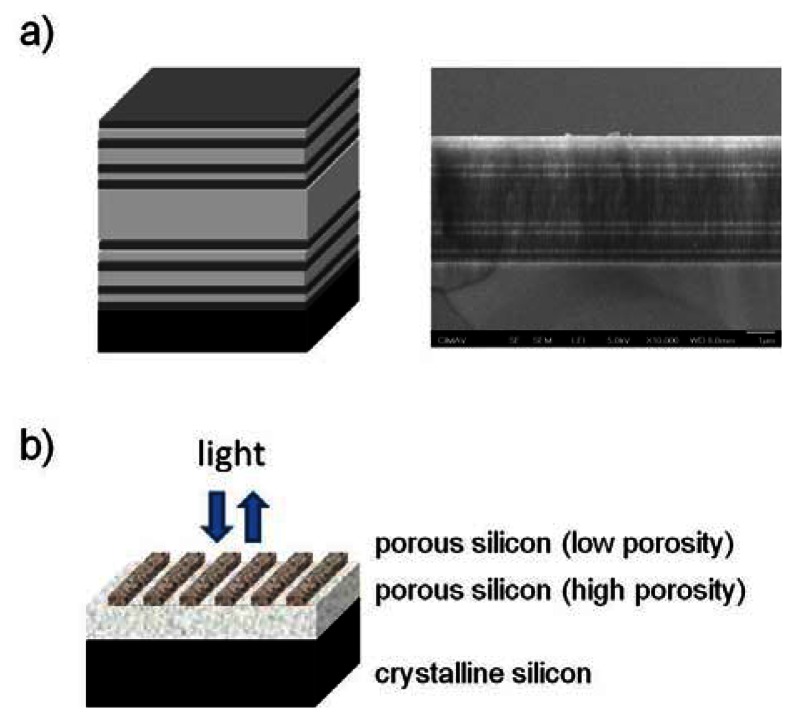
New porous silicon sensor lay-outs. (**a**) Schematic and SEM image of a Cantor structure. Image courtesy: V. Agarwal. (**b**) Schematic of a planar photonic crystal sensor.

Recently, fractal photonic crystals have been fabricated by electrochemical etching of silicon. They possess a deterministic nonperiodic modulation of the refractive index. Examples for 1D nonperiodic structure are Thue-Morse, Cantor, and Fibonacci [65–67]. A direct comparison of the optical sensor performance of periodic and deterministic nonperiodic porous silicon structures has been published by Moretti *et al.*, in 2007 [68]. They exposed porous silicon Bragg mirrors and Thue-Morse multilayers to VOCs and determined a higher sensitivity for the nonperiodic porous silicon structure. These results were explained by the narrower resonance peaks and the smaller amount of interfaces in the Thus-Morse structure in comparison to the Bragg stack. Appropriately designed porous silicon Cantor structures also showed higher sensitivity for the detection of silanization and further functionalization of the pore walls than porous silicon microcavities [69]. In Figure 9(a) a schematic and a SEM image of a Cantor structure etched into silicon are displayed. Another type of photonic structures so-called polybasic multilayers were suggested for the fabrication of highly sensitive porous silicon biosensors. Lv *et al.*, demonstrated their application to the detection of antigen-antibody interactions and DNA hybridization [70,71].

Besides these more exotic photonic structures classical 2D photonic crystal sensors based on porous silicon have been investigated for several years. The photonic band gap of 2D photonic crystal is observed for light propagation within the plane of periodicity. The fabrication of porous silicon 2D photonic crystals is more challenging than the production of a 1D photonic structure. Most porous silicon 2D photonic crystals were prepared from silicon-on-insulator substrates using standard lithography for the generation of an etching mask and reactive ion etching. Special care has to be taken for measuring the optical properties of these devices as the photons have to encounter the porous structure perpendicular to the pore axis. The demanding fabrication process for 2D photonic porous silicon structures has hindered its widespread application to sensor research. However, their high sensitivity makes them ideal candidates for the detection of very small amounts of analytes or even single molecule detection. The latter can be achieved by introduction of point defects in the lattice leading to a strong localization of light. Chow *et al.*, tested the sensitivity of a 2D photonic crystal microcavity to refractive index changes and determined a resolution of better than Δ = 0.001 [72]. Lee and Fauchet reported on the detection of a single latex sphere with a size which is comparable to viruses [73]. In addition they used a similar 2D photonic crystal sensor for monitoring glutaraldehyde and BSA binding to the internal sensor surface [73]. The extreme high sensitivity of 2D photonic crystal microcavity sensors was confirmed by studies performed in 2009. An amount of only 21 × 10^−18^ g of anti-biotin was detected overtrumping SPR based sensors [74]. The next generation of 2D photonic crystal sensors based on porous silicon possessed linear defects instead of point defects in the crystal lattice—2D photonic crystal waveguides [75]. By coupling micro- or nanocavities to silicon photonic crystal waveguides further advancements in the development of ultrasensitive optical sensors were made [76,77].

As already mentioned before the previously described 2D photonic crystal sensors suffer from time-consuming and expensive fabrication methods as well as complicated optical set-ups. In order to circumvent these high demands on the sensor device production and application, new concepts were developed focusing on the design of surface wave and planar 1D photonic crystal devices. Surface wave porous silicon sensors are obtained by etching a periodic porous silicon multilayer with a defect layer on top. The sensor is operated in Otto configuration which uses a single prism for coupling light into the structure. An evanescent wave is generated when light hits the sensor surface comparable to the well-known sensing principle of surface plasmon resonance sensors. Sensitive detection of ethanol vapor and amine grafting to the porous silicon surface wave sensors was demonstrated [78,79]. An example for a planar 1D photonic crystal is shown in Figure 9(b). The suggested planar photonic crystal sensor is composed of a periodic array of porous silicon bars on top of a porous silicon layer with a higher porosity and consequently a lower refractive index. This structure is characterized by the appearance of extreme narrow optical features—so-called Fano resonances—and can be used at normal incidence omitting complicated optical set-ups. Jamois *et al.*, have demonstrated the superior sensor performance for the detection of DNA hybridization [80]. Theoretical simulations predict that the sensitivity of these sensors could even be improved by periodic patterning of porous silicon multilayers [81].

## 4. Conclusions/Outlook

Porous silicon is a very attractive material for the design of tailor-made photonic crystal sensors. The ability to control the porosity and consequently the refractive index of the porous structure at will opens up countless possibilities for the construction of optical devices with the desired photonic properties. The fabrication of porous silicon sensors is relatively simple and cost-efficient allowing for mass-production. In combination with its very convenient and controllable surface chemistry porous silicon has to be taken seriously as competitor for established optical sensor devices like surface plasmon resonance based systems. The future will show if porous silicon will find its way from research labs into commercial applications.
